# Targeting Reactive Oxygen Species Metabolism to Induce Myeloma Cell Death

**DOI:** 10.3390/cancers13102411

**Published:** 2021-05-17

**Authors:** Mélody Caillot, Hassan Dakik, Frédéric Mazurier, Brigitte Sola

**Affiliations:** 1 Normandie University, INSERM, Université de Caen, F-14000 Caen, France; melody.caillot@unicaen.fr; 2 Université de Tours, GICC EA7501, CNRS, F-3700 Tours, France; hassan.dakik@mail.mcgill.ca (H.D.); frederic.mazurier@inserm.fr (F.M.)

**Keywords:** multiple myeloma, ROS, antioxidant enzyme, proteasome inhibitor, NOX, mitochondria, redox balance, OXPHOS

## Abstract

**Simple Summary:**

Multiple myeloma (MM) is a neoplastic disease of plasma cells, characterized by a complex array of clinical manifestations. Despite extensive efforts and progress in the care of MM patients, the disease is still fatal because of de novo or acquired resistance of malignant cells to standard chemotherapies. In turn, new therapies and/or combination therapies are urgently needed. Reactive oxygen species (ROS) are unstable and highly reactive chemical molecules, able to alter the main structural components of cells, such as proteins and lipids, and thus, modifying cell fates. ROS levels are tightly controlled in normal cells both for their production and degradation. In turn, an unbalance of the redox status might be exploited to induce cell death. This is indeed the case for myeloma cells even those that are resistant, opening new perspectives for refractory or relapsed MM patients.

**Abstract:**

Multiple myeloma (MM) is a common hematological disease characterized by the accumulation of clonal malignant plasma cells in the bone marrow. Over the past two decades, new therapeutic strategies have significantly improved the treatment outcome and patients survival. Nevertheless, most MM patients relapse underlying the need of new therapeutic approaches. Plasma cells are prone to produce large amounts of immunoglobulins causing the production of intracellular ROS. Although adapted to high level of ROS, MM cells die when exposed to drugs increasing ROS production either directly or by inhibiting antioxidant enzymes. In this review, we discuss the efficacy of ROS-generating drugs for inducing MM cell death and counteracting acquired drug resistance specifically toward proteasome inhibitors.

## 1. Introduction

Multiple myeloma (MM) is a plasma cell malignancy characterized by the accumulation of clonal cells in the bone marrow and the overproduction of monoclonal immunoglobulin causing the clinical features of the disease: hypercalcemia, renal failure, anemia and bone lesions, collectively known as CRAB symptoms. Moreover, plasma cells are prone to produce large amounts of immunoglobulins, causing an endoplasmic reticulum stress, the unbalance of redox homeostasis and the production of intracellular ROS [1]. The disease progresses from benign monoclonal gammopathy of undetermined significance (MGUS) and smoldering myeloma to overt aggressive MM and ultimately plasma cell leukemia. Over the past twenty years, MM patients outcome has been improved through the use of new drugs as well as new therapeutic approaches [2,3,4,5]. For elderly patients, not eligible to autologous transplantation, the use of proteasome inhibitors (PIs), immunomodulators (IMiDs), monoclonal antibodies, histone deacetylase (HDAC) inhibitors and more recently, check-point inhibitors and their various combinations have improved substantially MM patients survival. However, most MM patients are refractory or relapse (R/R) after one or more treatment regimens and the disease is still fatal [3,5,6].

The first-in-class PI, bortezomib (BTZ) and several second-generation PIs (carfilzomib (CFZ), ixazomib) are efficient drugs, currently used for newly diagnosed patients and R/R patients alone or associated with other agents [7]. By targeting subunits of the ubiquitin/proteasome system (UPS), the major cell regulator of protein degradation, PIs cause a proteotoxic stress that activates the apoptotic function of the unfolded protein response (UPR) [8]. Indeed, after UPS inhibition, misfolded proteins accumulate in the endoplasmic reticulum (ER) generating an ER stress and activating the UPR. The survival of MM cells is highly dependent on the UPR, and inefficient or prolonged UPR activation signals apoptosis [9,10,11]. Another important mechanism of BTZ cytotoxicity in MM cells is the generation of an oxidative stress that has been long considered as a byproduct of the ER stress. The production of ROS depends on ER-resident protein disulfide isomerase (PDI) and endoplasmic reticulum oxidoreduction (ERO1) enzymes as well as nicotinamide adenine dinucleotide phosphate (NADPH) oxidase (NOX) complexes and mitochondrial electron transport chain (ETC) [12]. The ERO1 flavoprotein consumes oxygen and generates hydrogen peroxide (H_2_O_2_). Under ER stress, the activity of these enzymes is increased and generates higher amounts of ROS and, in turn, an oxidative stress. Moreover, BTZ treatment induces an adaptative antioxidant response by depleting intracellular glutathione (GSH), activating CCAAT/enhancer binding protein (C/EBP)-homologous protein (CHOP) and transcription factor 4 (ATF4), two antioxidant transcription factors, and the nuclear factor-erythroid 2 p45-related factor 2 (NRF2), the key controller of detoxification genes [13].

Although BTZ and more generally PIs have achieved excellent therapeutic effects, innate resistance can be observed in drug-naive patients and acquired resistance may appear during the course of the treatment [14]. Indeed, during the progression of the disease, complex genetic alterations occur that contribute to the development of a resistant phenotype. The upregulation of the 20S proteasome subunits [15,16], the paradoxical downregulation of the 19S proteasome subunits [17], and the overexpression of efflux pumps [18] are among the main reported mechanisms of acquired resistance. However, the modulation of UPR, the alteration of apoptosis/autophagy signaling and metabolic changes contribute also largely to PI resistance. Indeed, a high glycolytic activity and/or the rewiring of glucose metabolism through the pentose phosphate pathway (PPP) and the serine synthesis pathway (SSP), an increased mitochondrial activity and fatty acid oxidation, are associated with PIs resistance [19,20,21]. A proteomic analysis comparing naive and R/R MM patients characterized four sets of relevant proteins including proteasomal proteins, apoptosis signaling proteins, proteins regulating the inflammation response, and factors regulating the redox status [22]. Moreover, the tumor microenvironment (TME) within the bone marrow facilitates tumor cells proliferation as well as drug resistance. This is due, at least in part, to a metabolic reprogramming of mesenchymal stromal and immune cells favoring the defense against ROS [23,24].

We face an apparent paradox. Due to their ability to secrete high amounts of immunoglobulin, MM cells undergo massive endoplasmic and oxidative stresses [25]. Although in accordance to the resulting high intracellular ROS levels, MM cells still respond to ROS inducers such as PIs and enter apoptosis. However, during the progression of the disease, within tumor cells and their TME, genetic and epigenetic changes occur that enable MM cells to escape death. Nevertheless, manipulating the redox status in MM cells including those that are PI-resistant may show therapeutic values. This review focuses on the redox landscape of MM cells and patients either naive or R/R and how manipulating the redox status in tumor cells could be effective in killing myeloma cells. We discuss the antimyeloma effects of drugs that buffer ROS or, by contrast, enhance ROS production, the latter counteracting the acquisition of PIs resistance.

## 2. Cellular ROS and Their Production

### 2.1. Generalities

The first non-mitochondrial consumption of O_2_ has been observed in granulocytes in 1959 [26]. This has been attributed to high H_2_O_2_ production during phagocytosis [27]. Later, the discovery of superoxide dismutase (SOD) showed the production of superoxide (O_2_^●^^−^) [28]. This production, derived from NOX activity, was subsequently demonstrated in granulocytes, settling the antibacterial function of ROS [29,30,31,32]. Nowadays, ROS are no longer only regarded as toxic byproducts, and their function limited to the innate response to infection, but are recognized as important secondary messengers involved in the regulation of many cell signaling cascades [33].

### 2.2. ROS Homeostasis

ROS are short-life electrophilic molecules arising from the partial reduction of O_2_ to O_2_^●−^ including free radicals that contain one or more unmatched orbital electrons, and non-radical ROS that can be chemically reactive and converted to radicals. The most abundant free radicals include O_2_^●^^−^, hydroxyl radical (HO^●^), and nitric oxide (NO). Non-radical ROS comprise H_2_O_2_, ozone (O_3_), peroxynitrate (ONOO^−^), and hydroxide (HO^−^) [34]. ROS homeostasis is maintained in cells by a tightly regulated balance between the production by oxidizing systems and the elimination by antioxidant systems. The alteration of this balance by increased ROS production or by decreased antioxidant activity leads to oxidative stress [35]. The exogenous ROS come from the environment, such as pollutants, smoke, radiation, temperature change, and xenobiotic compounds. The endogenous ROS are produced in the cell mainly by NOX, ETC, ER, xanthine oxidase (XDH), nitric oxide synthase (NOS), lipoxygenase (LOX), cyclo-oxygenase (COX), and other metabolic activities such as β-oxidation in peroxisomes, prostaglandin synthesis, and cytochrome P450 detoxification reactions [34,36]. To protect against harmful ROS effects, cells have retained during evolution antioxidant systems enabling their detoxification. The antioxidants include a variety of enzymes, such as SOD, catalase (CAT), glutathione peroxidases (GPX), peroxiredoxins (PRDX), and glutaredoxins (GLRX), as well as non-enzymatic systems, such as vitamins A, C, D, E, β-carotene, and ubiquinone [37].

### 2.3. ROS Targets

At low levels, ROS can react in a reversible manner with various elements. In particular, H_2_O_2_, a transient secondary messenger, oxidizes the reactive cysteine residues of redox-sensitive proteins and alters their function. Similar to phosphorylation by kinases, alteration of the thiol group modifies the activity of proteins in an inhibiting or activating way [38]. In contrast, at high levels, ROS irreversibly reacts with heme iron, as well as the carbon of specific amino acids (arginine, lysine, proline, and threonine) and nucleosides [39]. DNA oxidation by ROS is treated by activation of the nucleotide excision repair and base excision repair pathways [40]. Some oxidative damages, such as amino acid carbonyl groups, are not repairable. This leads to the degradation of oxidized macromolecules by the proteasome [41] or the autophagosome [42]. The list of proteins physiologically regulated by ROS increases regularly, and includes variable-pathway proteins: tyrosine and serine/threonine phosphatases, tyrosine and serine/threonine kinases, transcription factors, HDACs, heat-shock proteins (HSPs), caspases, metalloproteinases, PIs, metabolic enzymes, and ion channels [43].

### 2.4. Physiological ROS Functions

ROS are crucial in multiple signaling pathways by reacting directly with proteins, transcription factors, and genes. Thus, ROS are involved in a variety of processes, from inflammation and pathogen elimination, to adaptation to hypoxia, growth, and cell differentiation.

#### 2.4.1. Cell Signaling

The activation of tyrosine kinase (TK) receptors by growth factors induces cell proliferation, differentiation, migration, and survival. The phosphorylation cascade that follows the binding of a growth factor to its receptor may be attributed, at least partially, to ROS production [44]. For example, activation of epidermal growth factor receptor (EGFR) by its ligand induces ROS production by NOX [45]. The ROS produced, mainly H_2_O_2_, alter the activity of redox-sensitive target proteins by modulating specific reactive cysteine residues. So, they inhibit tyrosine phosphatase proteins on one side [46], and activate EGFR on the other [47], leading to an increased TK activity and signal amplification. This transient ROS signaling can be reverted by the PRDXs that reduce oxidized cysteines in a glutathione- or thioredoxin-dependent way [48].

#### 2.4.2. Hypoxia Adaptation

Hypoxia-inducible factors (HIFs) compose a family of dimerized transcription factors, involved in the cellular adaptation to low oxygen levels. In normoxia, prolyl-hydroxylases (PHD) hydroxylate two proline residues in the HIF-α subunits inducing their recognition by the von Hippel-Lindau (VHL) tumor suppressor protein with an ubiquitin ligase activity, and their degradation by the proteasome. In hypoxia, their stability is mediated by the absence of oxygen as a co-factor of PHD, and by mitochondrial ROS. PHDs are further inhibited, allowing the accumulation of HIF-α subunits, their hetero-dimerization with HIF-β, their translocation to the nucleus, and the transcription of their target genes, such as those involved in cell metabolism [49,50,51]. In addition, mitochondrial ROS can induce transcription by direct oxidation of DNA. Such oxidation of the vascular endothelial growth factor (VEGF) promoter facilitates the binding of HIF-1α, for example [52].

#### 2.4.3. Retro-Control and Regulation of Antioxidants

NRF2 regulates the expression of antioxidant genes by recognizing antioxidant response elements (ARE) in their promoters [53]. NRF2 localized in the cytoplasm, is attached to the Kelch inhibitory protein-like ECH-associated protein 1 (KEAP1). The increase in ROS level modifies a KEAP1 cysteine residue that detaches from NRF2 allowing it to migrate into the nucleus. This similarly happens to the transcription factor FOXO4, whose intermolecular oxidation of reactive cysteines allows the bond with the activator couple EP300-cAMP-response element binding protein (CREB) binding protein (p300/CBP). This induces its activation and transcription of antioxidants such as SOD2, CAT, and PRDX, and leads therefore to decreased ROS levels [38,54]. In addition, the oxidation of ataxia telangiectasia mutated (ATM) kinase cysteines by ROS induces a flow of glucose to the PPP allowing the synthesis of NADPH essential for antioxidants as well as DNA repair [55].

### 2.5. Oxidants

#### 2.5.1. Mitochondria

The mitochondria is generally regarded as the major producer of cellular oxidants. It mainly produces ATP depending on O_2_ [56]. During this process, an electron leak induces the generation of O_2_^●^^−^, a secondary product [33]. The ETC contains five protein complexes localized in the intermembrane space, the complexes I–IV and the ATP synthase, as well as two soluble factors that function as electron acceptors: the cytochrome c (CYCS), which is a protein, and the coenzyme Q10, which is a lipid known as ubiquinone [57]. First, the complexes I and II receive electrons from the nicotinamide adenine dinucleotide (NADH) and succinate, respectively, and transfer them to the complex III via the coenzyme Q10. Next, the complex III transfers the electrons to CYCS, which in turn transfers them to complex IV (cytochrome reductase) reducing O_2_ to H_2_O [58]. During this electron transfer, a pH gradient occurs allowing ATP synthesis by the ATP synthase. However, 1–2% of electrons escape the ETC and react directly with O_2_ reducing it to O_2_^●^^−^ [59]. Superoxide can be generated at the complex III level, during the Q-cycle of electron transfer from coenzyme Q10 to CYCS [60]. The coenzyme Q10 accepts two electrons from the complex I or II and is reduced to QH2, which yields them to CYCS in two stages at the Qo site of the complex I near the intermembrane space. After the transfer of the first electron, the oxidized coenzyme Q (QH•) can react with O_2_ to generate O_2_^●^^−^ released in the intermembrane space. QH• is usually re-reduced to QH2 within the Qi site of complex III near the matrix. The production of O_2_^●^^−^ at the level of complex III is particularly high under hypoxia where H_2_O_2_ accumulates in the intermembrane space. It diffuses to cytosol triggering for example, PHD-dependent redox inhibition, then stabilizing HIF-α [50].

In addition, O_2_^●−^ may also be produced by mitochondrial matrix metabolic enzymes such as glycerol-3-phosphate dehydrogenase 2 (GPD2), electron transfer flavoprotein dehydrogenase (ETFDH), 2-oxoglutarate dehydrogenase (OGDH), and pyruvate dehydrogenase complex (PDC) [33,61].

#### 2.5.2. NADPH Oxidases

The NOX2 complex, with CYBB (gp91^phox^) as the catalytic subunit encoded by the *CYBB* gene, is the first complex discovered at the neutrophil membrane [32]. Later studies have shown that a variety of ligands such as tumor necrosis factor (TNF), platelet-derived growth factor (PDGF, angiotensin I, and EGF) can induce the generation of intracellular ROS in non-phagocytic cell even in the absence of NOX2 [62,63,64]. This production led to the discovery of the NOX1 complex [65]. Comparative analyses identified five additional complexes, NOX3-5, and DUOX1-2 [66,67,68] Although similar, each complex has distinct structural, biochemical, and cellular localization features [34]. Today, NOX-derived ROS are known to be involved in cellular signaling besides their antibacterial role [69]. These transmembrane flavoprotein enzymes are the only ones to physiologically produce ROS and, next to mitochondria, form an important source of ROS in cells [70]. According to the needs, several regulatory ways exist to limit both the expression and activity of ROS over time and space. Following activation, they catalyze the electron transfer of NADPH through a biological membrane to O_2_ from the different intra- and extracellular compartments and reduce it to O_2_^●^^−^, which is converted into H_2_O_2_ by SODs or directly, in the case of NOX4 and DUOX [34].

### 2.6. Antioxidants

SOD, CAT, GPX, and PRDX form the first-line of cellular antioxidant defense by metabolizing O_2_^●−^ and H_2_O_2_. SODs are the only enzymes that eliminate O_2_^●−^ by catalyzing its dismutation into H_2_O_2_ and O_2_. While SOD1 and SOD3 depend on copper for their activity, SOD2 is dependent on manganese. They are distinguished by their cellular location: SOD1 is located in the cytoplasm, the mitochondrial intermembrane space, and the nucleus, SOD2 is located in the mitochondrial matrix, while SOD3 is extracellular [71]. CAT catalyzes the transformation of H_2_O_2_ into H_2_O and O_2_. It is expressed in all cell types with the exception of erythrocytes [72] and vascular cells [73]. There are eight human GPX, among which five are selenoiproteins (GPX1-4 and GPX6), whereas the other three (GPX5 and GPX7-8) depend on thiol instead of selenol [74]. Selenoiproteins and thiol peroxidases promote the two-electron reduction of H_2_O_2_ to H_2_O using GSH as a reducing agent [75]. They are associated with glutathione reductase (GSR), which catalyzes oxidized glutathione reduction (GSSG) using NADPH as a reducing agent. GPX1 and GPX2 are mainly cytoplasmic, whereas GPX3, GPX5, and GPX6 are extracellular [74]. Splicing of GPX4 results in three isoforms having cytoplasmic, nuclear, or mitochondrial localizations whereas GPX7 and GPX8 are respectively in the membrane and cisternal space of ER [74].

Like GPX, PRDX (1–6) are thiol peroxidases containing reactive cysteines that allow them to eliminate H_2_O_2_ to produce H_2_O [76]. They function together with the thioredoxin system [75]. In humans, six PRDX enzymes are expressed and differ by their cellular locations: PRDX1, PRDX2, and PRDX6 are cytoplasmic, PRDX3 is restricted to mitochondria, PRDX4 is localized in ER, whereas PRDX5 is in the cytosol, mitochondria, and nucleus [76].

The thioredoxin system includes thioredoxins (TXN and TXN2), and thioredoxin reductases (TXNRD1-3), as well as NADPH as a cofactor. TXN is cytoplasmic and nuclear, whereas TXN2 is mitochondrial [77]. Among TXNRDs, TXNRD1 is cytosolic, TXNRD2 is mitochondrial and TXNRD3 is both cytoplasmic and nuclear [78]. Thioredoxins are small thiol-disulfide oxidoreductases that provide the electrons needed for H_2_O_2_ removal by PRDX. In turn, TXNRD catalyzes the recycling of oxidized thioredoxins using NADPH as a reduction agent [76,79].

Finally, the glutaredoxin system includes GLRX, GSR, as well as NADPH and GSH as cofactors. GLRX catalyzes oxidation-reduction reactions of GSH-disulfides, deglutonylation of S-glutathionylated proteins, and reduction of TXNs, in the absence of TXNRD, using GSH as the reducing agent. Human cells express four glutaredoxin paralogs (GLRX, GLRX2-3, and GLRX5) that differ in their localization. GLRX and GLRX3 are cytoplasmic, whereas GLRX2 and GLRX5 are mitochondrial [80]. As with GPX, GSR catalyzes oxidized glutathione reduction (GSSG) using NADPH as a reduction agent.

**Figure 1:**
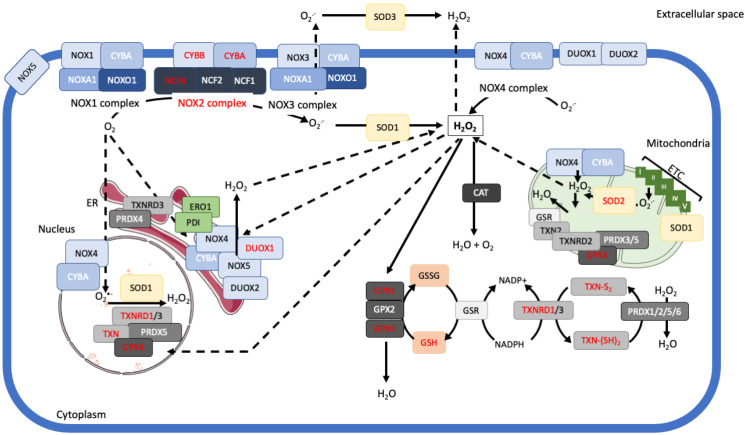
Cell mechanisms of redox regulation. The two key reactive oxygen species (O_2_•^−^ and H_2_O_2_), that serve as intracellular signaling molecules, are mainly produced by the transmembrane NOXs and the mitochondria electron transport chain (ETC). NOXs are found at different localizations (cell membrane, organelles, nucleus) thereby contributing to local generation of ROS. Apart from NOXs and the ETC, H_2_O_2_ is also produced by various oxidases present in the ER as well as by several SODs. ROS are sensors of environmental changes and stresses and permit cells to adapt these changes. In turn, the redox status of cells is finely tuned by various antioxidant systems. ROS detoxification involves two main systems: non-catalytic small molecules that directly scavenge ROS including glutathione (GSH) and catalytic antioxidant enzymes. Catalytic enzymes that scavenge O_2_•^−^ include the SODs, each catalyzing the conversion of O_2_•^−^ into H_2_O_2_ and collectively, controlling NOXs activity. SOD2 (in red) is overexpressed in MM cells (see Section 3). Enzymes that scavenge H_2_O_2_ include catalase (CAT) which converts H_2_O_2_ into H_2_O + O_2_ as well as peroxiredoxin (PRDX) and glutathione peroxidase (GPX). Reduction of H_2_O_2_ by PRDX1-5 is at the expense of oxidized thioredoxin (TNX) which leads to thioredoxin with intramolecular disulfide bridge (TXN-(SH)_2_ ➛ TXN-S_2_), whereas PRDX6 requires GSH rather than TXN). Reduction of H_2_O_2_ by GPX also requires TXN but, for that, the thiol group between two cysteines from two GSH molecules yields an intermolecular disulfide bridge after reduction (GSH ➛ GSSG). TXN and TXNRD1, GPX1 and GPX4 (in red) are overexpressed in myeloma cells compared to normal plasma cells [81]. Moreover, NOX2 (CYBB) is the only catalytic NOX subunit expressed in myeloma cells together with NCF4 (p40^phox^) and CYBA (p22^phox^) regulatory subunits. In turn, only the NOX2 complex is functional in those tumor cells [81].

The dynamics of ROS production as well as their elimination through the antioxidant systems are schematized in Figure 1.

## 3. The Redox Landscape in Multiple Myeloma 

**Figure 2:**
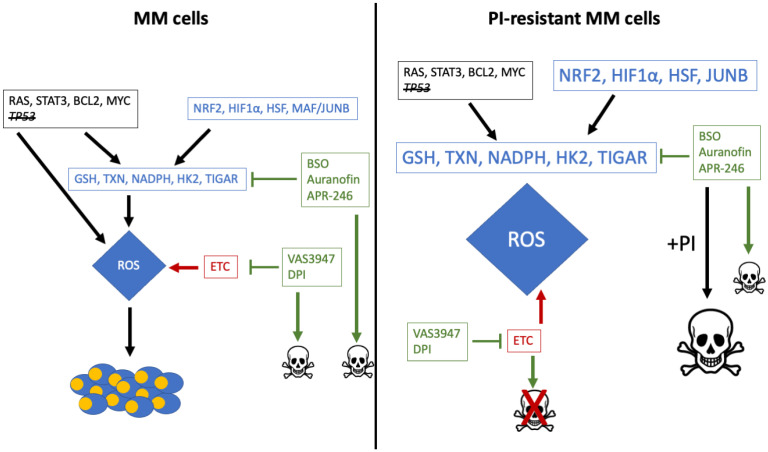
Schematic representation of MM cells adaptation to elevated intracellular ROS and ROS-mediated PIs resistance. In MM cells, activation of keys oncoproteins (RAS, STAT3, BCL2, MYC) and inactivation of *TP53* result in an increase of intracellular ROS. To avoid entering apoptosis, cells adapt their antioxidant capacities and enhance the transcriptional activity of factors targeting GSH- and TXN-dependent enzymes and detoxifying systems. The decrease of GSH and the inhibition of antioxidant enzymes (TXN, TXNRD1) by gold compounds (auranofin) or L-buthionine sulfoximine (BSO), an irreversible inhibitor of the glutamate-cysteine ligase (GCL or γ-glutamylcysteine synthetase), one enzyme of GSH synthesis, as well as the inhibition of mitochondrial respiration complex I with diphenyleneiodonium (DPI), trigger MM cells apoptosis (see chapter 4). Due to genetic and epigenetic changes occurring during PI-treatment, drug resistance rises. In PI-resistant cells, the targeting of antioxidant enzymes (TXN and TXNRD1 inhibitors), or the pool of GSH (BSO and APR-246) is still efficient and restores drug sensitivity. The association of auranofin and BTZ exacerbates the level of ROS and is more efficient for inducing MM cell death.

Cancer cells including myeloma cells contain higher levels of ROS than their normal counterparts and this high level of ROS sustains the malignant phenotype [82]. Indeed, it is well-established that ROS initiates the tumorigenesis process, fuel cell proliferation and survival, and influence the tumor microenvironment during the progression of the disease. To avoid entering senescence or dying, MM cells increase their GSH content as well as enhance the activity of TXN and TXNRD1 enzymes. Adaptation to this chronic oxidative stress necessitates a metabolic reprogramming that culminates with enhanced oxidative glycolysis, glutaminolysis, and fatty acid oxidation although MM cells still use mitochondrial respiration [83]. The molecular basis of MM cells adaptation to oxidative stress as well as ROS-mediated PI-resistance is described below and schematized in Figure 2.

### 3.1. Activation of Antioxidant Transcription Factors

Multiple metabolic programs are controlled at the level of gene transcription by transcription factors. HIF-1α is stabilized by hypoxia; it regulates the expression of GSH-based genes under hypoxic conditions and exerts antioxidant effects by decreasing mitochondrial ROS production through glucose catabolism [82,84]. HIF-1α is constitutively activated and stabilized in MM cells [85,86,87,88]. In BTZ-resistant cells, HIF-1α is even more expressed leading to an increased glycolysis that supports resistance [19].

The heat shock factor 1 (HSF1), the master regulator of heat shock response, and its transcriptional targets are associated with MM patient outcome and necessary for MM cells survival [89]. HSF1 targets the ROS scavenger PRDX4 that is highly expressed in MM cells [81] and in turn, controls cellular H_2_O_2_ levels. HSF1 inhibitors synergize with BTZ to induce MM cells death from MM patients including those belonging to adverse prognosis groups or in relapse [90].

NRF2 is the master regulator of the ROS stress response in MM cells [91]. In the molecular subgroup of MM cells having the t(4;14) translocation, overproducing MMSET, a histone methyltransferase, associated with a poor prognosis [92], a small nucleolar RNA (snoRNA) called ACA11, inhibits NRF2, increases ROS levels, and drives MM cells proliferation [91]. In response to cellular stress, NRF2 activates stress-protective genes such as NOX, heme oxygenase 1 (HO1), or the autophagy receptor sequestosome 1 (SQSTM1 or p62) [93]. In CFZ-resistant cells, NRF2 is upregulated and activated, which promotes enhanced expression of NRF2 target genes including GSR and GPX4. Moreover, in agreement with NRF2 function in facilitating the production of NADPH cofactor by directing the carbon flux through the PPP, NADPH levels are high in CFZ-resistant MM cells, disrupt the redox homeostasis, and affect also fatty acid oxidation [94]. In response to high ROS levels, NRF2 stimulates the expression of Kruppel-like factor 9 (KLF9). Then, KLF9 causes accumulation of ROS by suppressing *TXNRD2* transcription [95]. It is worth noting that in MM cells, BTZ- and CFZ-resistance is correlated to high levels of KLF9 [96].

The AP-1 transcription factors family comprises several proteins that function through their dimerization. They exert antioxidant effects via the induction of genes encoding ROS scavengers, synthesizing GSH and metabolizing pro-oxidant xenobiotics [82]. Among them, JUNB and MAF are important for MM pathophysiology. MAF (c-MAF or MAFB) is a basic leucine zipper-containing oncoprotein expressed in almost 50% of MM having or not the t(14;16) and t(14;20) transactivating translocations [97]. Although, to our knowledge, a role of MAF in PI-resistance has not been reported so far, JUNB, specifically induced by the TME, controls BTZ-resistance by regulating genes of the oxidative metabolism and the mitochondrial respiration [98].

Five proteins belong to the nuclear factor (NF)κB family: NFκB1 (p50 and its precursor p105), NFκB2 (p52 and its precursor p100), RELA (p65), RELB, and c-REL [99]. Their transcriptional activities are mediated by their various associations that generate homo- and heterodimers. The activation of the NFκB pathway by external or internal stimuli occurs through the canonical or alternative pathways [99]. In MM cells, an increased cytokine stimulation from the TME as well as mutations of genes encoding NFκB proteins and their regulators (*NFKB1/2*, *TRAF2/3*, *TACI*, *MAP3K14*) lead to the constitutive activation of the pathway [100,101,102]. Genes coding for CAT, GPX1, and SOD1/2 are controlled by NFκB members [82].

During the development of MM disease, an increased accessibility of chromatin is observed favoring global histone acetylation and chromatin activation [103]. Recent data obtained using a multi-epigenomic approach showed that MM cells are characterized by an aberrant chromatin activation [104]. Among the target genes upregulated by this process, are members of the NFκB and p53 signaling pathways, and response to oxidative stress. Among them, *TXN* is activated and the protein overexpressed due to de novo activation of a distant enhancer.

### 3.2. Activation of Oncoproteins That Stimulate ROS Production

Mutations of NRAS, KRAS, BRAF oncogenes are found in up to 50% of newly diagnosed MM patients and associated with a poor prognosis. Most of them cause the constitutive activation of the RAS/MEK/ERK survival signaling pathway and modulate PI sensitivity [105,106]. Moreover, mitochondrial metabolism and mitochondrial ROS generation are essential for RAS-induced cell proliferation and tumorigenesis [107].

Almost all MM cells express one of the three cyclin D-type proteins. Thus, it has been proposed that the dysregulation of one of the CCND genes encoding cyclins D is an unifying event for MM pathogenesis [92]. Cyclins D in dimer with their catalytic partners, the cyclin-dependent kinase (CDK)4/6, controls the early phase of the cell cycle i.e., the progression within the G1 phase and the G1-to-S phase transition. However, depending on their subcellular localization, activation or/and associated partners, cyclins D possess various cell functions that are not necessarily redundant [108]. Regardless the t(11;14) translocation that activates the CCND1 gene, cyclin D1 is present in almost 50% of MM cells. In the remaining cases, cyclin D2 and cyclin D3 are expressed as a consequence of FGFR3/MMSET or MAF deregulations corresponding to t(4;14) and t(14;16) or t(14;20) translocations, respectively [92]. In MM cells, cyclin D1 increases NOX2 activity and produces ROS, thereby disrupting the redox balance and modifying drug sensitivity [109]. Moreover, cyclin D1 acts as a cofactor of HIF-1α and transactivates the HK2 gene in turn, enhancing the oxidative glycolysis [110]. Finally, cyclin D1 facilitates the shift from OXPHOS to glycolysis by competing with hexokinase 2 (HK2) for voltage-dependent anion channel (VDAC) binding and metabolites/ATP entrance into the mitochondria [111].

Fibroblast growth factor (FGF) and FGF receptors (FGFR) are expressed on MM cells suggesting that an autocrine signaling contributes to myeloma proliferation. Moreover, this autocrine signaling protects MM cells from ROS-induced apoptosis. Indeed, FGF blockade by FGF trapping or tyrosine kinase inhibitor induces a GSH depletion and mitochondrial ROS production [112]. This oxidative stress is the consequence of c-MYC proteasomal degradation. Moreover, these findings were also observed in BTZ-resistant cell lines and in primary cells obtained from R/R patients [112]. c-MYC is an oncoprotein and transcription factor that drives the progression from MGUS to MM. MYC activation relies on translocation involving the IGH locus and gain and amplification at 8q24 [113]. c-MYC is a major player in the regulation of glycolysis and glutaminolysis but its role in the control of redox homeostasis is less documented [114].

MCL1, BCL2, and BCLXL are antiapoptotic proteins overexpressed in MM cells [115] and associated with a bad prognosis [116]. At the outer mitochondrial membrane, they prevent death by sequestering proapoptotic BH3-only activators such as BIM, PUMA, BID. The BH3-only sensitizers, such as NOXA, BAD release activators by binding antiapoptotic proteins. Then, the BAK and/or BAX effectors activate the subsequent steps of apoptosis [116]. A number of reports attribute the death-inducing activity of BCL2 family proapoptotic members to the modulation of cellular redox metabolism [117]. The samples belonging to the MM subgroup with a t(11;14) and expressing cyclin D1 respond well to venetoclax, a BH3-mimetic [118]. They have reduced mitochondrial respiration due to reduced complex I and II activities, the latter being linked to the generation of ROS [119]. Conversely, an elevation of oxidative phosphorylation (OXPHOS) promotes venetoclax resistance.

B7-homolog 3 (B7-H3) or CD276 is a member of the B7 family of checkpoint molecules. B7-H3 is highly expressed on MM cells, promotes glucose metabolism, and participates in drug resistance. Indeed, B7-H3 activates the JAK/STAT3 pathway through the redox-mediated oxidation and activation of SRC and the degradation of SOCS3 [120].

Mucin 1 (MUC1) is a glycoprotein aberrantly expressed by MM cells and the MUC1 C-terminal oncoprotein (MUC1-C) is involved in cell proliferation and survival [121]. The treatment of MM cells with GO-203, an inhibitor of MUC1 homodimerization, induces ROS production and a significant downregulation of the TP53-induced glycolysis and apoptosis regulator (TIGAR). The production of ROS is due to the depletion of both NADPH and GSH [122,123]. Moreover, the GO-203/BTZ combination is effective for killing BTZ-resistant MM cells [122].

### 3.3. Increase of Antioxidant Defenses

MM cells increase their metabolic demand when the disease progresses and produce high levels of ROS. In turn, MM cells highly depend on antioxidant systems: non catalytic small molecules of ROS scavengers such as GSH, catalytic enzymes (SOD1/2) that scavenge O_2_^●^^−^, and those (CAT, PRDX, GPX) that scavenge H_2_O_2_. Intracellular GSH level determines the sensitivity of MM cells to BTZ [93]. The cysteine-glutamate antiporter Xc− imports cystine, regulates GSH levels and consequently, BTZ-response and -resistance. Increased GSH level abolishes BTZ-induced death, whereas reciprocally the blocking of Xc− potentiates PI-mediated cell death [93].

By contrast with GPX1/4, GPX3 is not expressed in MM cell lines or patients [81]. The transcription of GPX3 is controlled by the methylation of its promoter. Importantly, this epigenetic silencing of GPX3 is associated with MM disease progression and adverse prognosis for patients [124].

Antioxidant genes coding for TXN and TXNRD1 are overexpressed in myeloma patients and MM cell lines when compared to normal plasma cells [96,125,126,127]. GLRX2/3 and TXN are overexpressed in MM molecular subtypes with adverse prognosis (namely PR, MS, HY), whereas PRDX6 and SOD1 expression are associated with a shorter overall survival [81,128]. A high expression of either SOD1 or SOD2 contributes also to BTZ resistance [129,130]. Moreover, a multi-epigenomics approach demonstrated that an aberrant chromatin activation is a common feature of MM cells. Among the targeted genes activated by this process are genes involved in the response to oxidative stress including TXN [104].

### 3.4. Metabolism Rewiring

**Figure 3:**
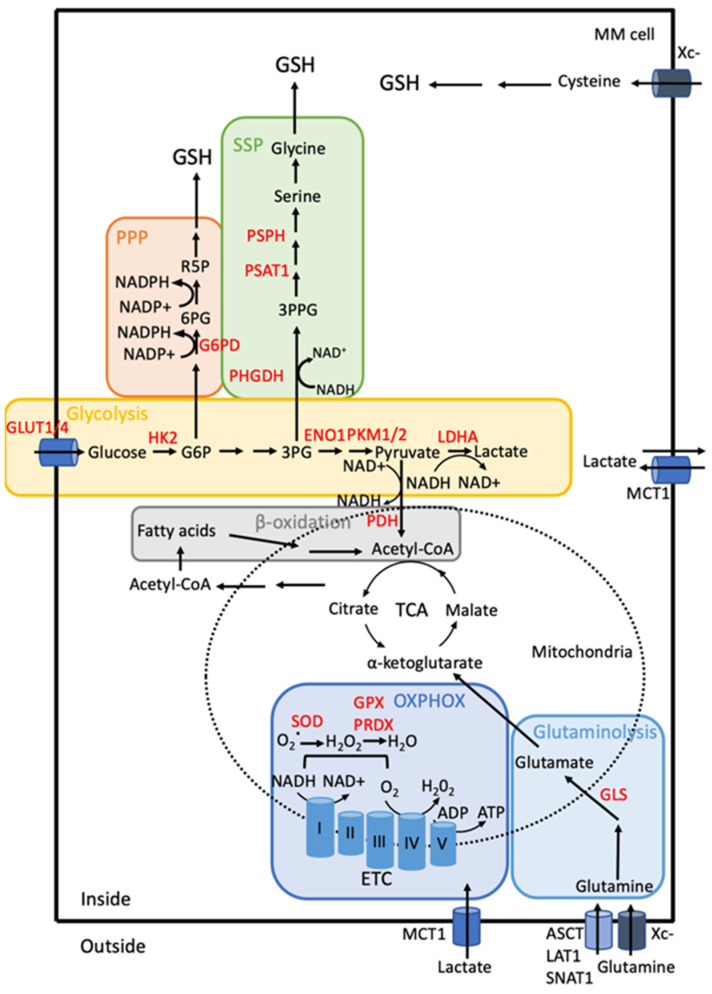
Schematic representation of metabolism rewiring in MM cells. MM cells are glycolytic. GLUT1/4, glucose transporters. The levels of GLUT1/4 are high and allow an elevated glucose uptake. Intracellular glucose is transformed into lactate by oxidative glycolysis. This multi-step catabolism is controlled by several glycolytic enzymes all overexpressed in MM cells such as HK2, PFK, PKM2, LDHA [19,110,137,138]. The end-product of glycolysis, lactate is secreted by MM cells. However, MM cells take up exogenous lactate produced by the surrounding TME. Lactate enters cells through the MCT1/4 transporters and fuels OXPHOS fully performing in MM. Glutamine synthase is lacking in MM cells, therefore MM cells are dependent on glutamine uptake. By contrast, glutaminase (GLS) expression is increased [139]. PDH is a gatekeeper enzyme that converts pyruvate into acetyl-CoA and regulates the mitochondrial metabolism. PDK1 is a serine/threonine kinase that negatively regulates PDH, its inhibition induces MM cell death [133]. The serine metabolism is also involved in MM cell adaptation to BTZ treatment. Phosphoglycerate dehydrogenase, PHGDH, the first limiting enzyme of the SSP pathway that converts 3-PG into PHP is elevated and increases GSH synthesis in BTZ-resistant cells [20,137]. G6PD, the first rate-limiting enzyme of the PPP, converting G6P into 6PG, is elevated in MM cells and associated with an exacerbated flux [140]. Associated with increased mitochondrial biomass and function, MM cells have higher concentrations of ATP, NADH/NADPH ratio and mitochondrial membranes are hyperpolarized, all these changes leading to impaired drug response and resistance [129,141]. β-oxidation participates in the myeloma metabolism changes [19]. The abbreviations used in the Figure 3 are gathered at the end of the text.

Compared to normal plasma cells, MM cells have enhanced metabolic activity including glycolysis, serine synthesis pathway (SSP), glutaminolysis, fatty acid or β-oxidation all benefiting tumor cells against oxidative damage (Figure 3) [131].

MM cells also utilize OXPHOS and glycolysis/glutaminolysis compensate each other [132]. Proteasome inhibition results in even a higher glycolytic activity [19,20,22]. This glycolytic activity contributes significantly to the generation of reducing equivalent, such as NADPH, the most important oxidizing agent, regenerated from NADP+ when malate is converted into pyruvate through the tricarboxylic acid (TCA) cycle. The generation of NADPH makes MM cells more tolerant to UPS inhibition [22]. The reduction of lactate dehydrogenase A (LDHA) activity and the inhibition of pyruvate dehydrogenase kinase 1 (PDK1), the serine/threonine kinase that negatively regulates PDH, lead to an elevation of NADH/NADPH ratio, and an increase of ROS production [133]. Lactate produced by MM cells as the last metabolite of glycolysis is transported through monocarboxylate transporters (MCT1/4) and fuels OXPHOX [134].

Deregulated metabolic processes involved in protein biosynthesis, GSH synthesis, malate/aspartate shuttle, metabolism of purines, pyrimidines, amino acids, and the TCA cycle also consistently change as observed in PI-resistant cells [21]. The rewiring of glucose metabolism through the PPP and SSP is associated with PIs resistance [19,20,21,135]. These pathways lead to increased antioxidant capacities through the recovery of GSH, higher redox homeostasis, and maximum generation of NADPH [136].

The integrity of mitochondrial function is necessary for limiting oxidative stress. A reduced efficiency of ETC is associated with alteration of the PPP, increases GSH levels in MM cells [142]. Moreover, PI-resistant MM cells display global changes of mitochondrial metabolism [142]. MM cells overexpress a mitochondrial biogenesis signature and this signature is even exacerbated in R/R patients [143]. The accompanying increase of the mitochondrial mass is recognized as an adaptive response to treatments [22,129]. MM cells resistant to BTZ have high concentrations of ATP, NADH/NADPH ratio, and a hyperpolarization of the mitochondrial membrane [144]. These biomarkers are also predictors of MM sensitivity to venetoclax, a BH3-mimetic that targets the BCL2 antiapoptotic molecule [119]. An elevated mitochondrial mass might be associated with drug resistance irrespective of its mechanism of action.

### 3.5. Impact of ROS Production on the Immune Tumor Microenvironment

MM progression is tightly associated with a dynamic modeling of its surrounding environment [145]. The bone marrow niche in which MM develops is a complex system comprising the vascular and endosteal niches as well as the immune microenvironment [146]. Several recent reviews focused on the role of vascular and endosteal niches in the development of MM pathology and mechanisms of drug resistance (e.g., [147,148,149]). The oxidative stress imposed by ROS production in the bone marrow environment, causes an immunosuppressive state and impairs endogenous signaling of immune cells [146]. ROS are produced by MM cells themselves and also by tumor-associated macrophages (TAMs) that infiltrate the BM compartment [150]. TAMs are polarized toward a M2-like phenotype that is associated with immunosuppressive functions [151]. ROS produced by M2 macrophages recruit regulatory T cells (Tregs) to the TME. Tregs are highly vulnerable to ROS in the TME and Tregs achieve strong suppressor function through an oxidative stress-dependent apoptosis [152]. Myeloid-derived suppressor cells (MDSCs) are a heterogeneous population of myeloid cells with immunosuppressive competence [153]. Although, they could achieve immunosuppression using various mechanisms [153], they induce Tregs in the TME [154]. Moreover, MDSCs produce ROS in a NOX2-dependent manner and released ROS suppress T-cells proliferation thereby enhancing immunosuppression [155]. Tumor-infiltrating lymphocytes (TILs) comprise cytotoxic and helper lymphocytes as well as natural killer (NK) cells [156]. Within the TME, it appears that T cells are exhausted and senescent exhibiting aberrant function [157] whereas the cytotoxicity of NK cells toward MM cells is impaired [158]. As a whole, though ROS production, MM cells evade the immune system through two concomitant strategies: impairing immune recognition and enhancing the immunosuppressive state of TME [159].

## 4. Unbalancing ROS in Multiple Myeloma

The rational for manipulating the redox status of MM cells to induce their death and/or to alleviate their resistance is an old concept [160]. This is based on the observation that the oxidative metabolism in myeloma vs. normal plasma cells is fundamentally different. Theoretically, either oxidant or antioxidant treatment could exacerbate an oxidative stress and in turn, cell death. Indeed, myeloma cell death is obtained either by decreasing ROS levels or by increasing them (Table 1). ROS are synthesized in MM cells at least in part by the NOX complex activity [109]. Moreover, compared to normal bone marrow plasma cells, patients with MGUS, or a smoldering myeloma, NOX2 (encoded by *CYBB*) is the only catalytic subunit expressed in MM patients along with the p40^phox^ (*NCF4*) and p22^phox^ (*CYBA*) regulatory subunits [81]. Although a pan-NOX inhibitor, VAS3947 has a strong anti-MM activity, it shows adverse effects when combined with BTZ [81].

The effects due to pro-oxidant strategies are more documented. Increasing the intracellular ROS level can be achieved either by their direct production or by inhibiting antioxidant defenses.

### 4.1. Drugs That Target Glutathione Metabolism

*TP53* mutations/deletions are present in most cancers and associated with resistance to therapies. In turn, molecules acting downstream of p53, inducing the transcription of p53 targets such as p21^CIP^, BAX, PUMA, and NOXA and reactivating cell death have been selected. In MM cells, *TP53* mutations/deletions are rare but increase with disease progression and associate with a bad prognosis. p53-modulating agents such as CP-31398, PRIMA-1 (p53 reactivation and induction of massive apoptosis-1), or APR-246 (PRIMA-1^Met^) induce ROS-mediated apoptosis of MM regardless the *TP53* status [161,162,163,164,165]. At the molecular level, APR-246 induces ROS production by depleting GSH cell content [161,164]. Interestingly, APR-246 synergizes BSO, an irreversible inhibitor of the GSH synthesis. Another report indicated that caffeic acid phenetyl ester (CAPE), a phenolic compound, induces apoptosis through an oxidative stress caused by glutathione depletion [166].

**Table 1:** Drugs targeting redox metabolism in MM preclinical models.

Effects on ROS Level	Drug/Combination	Effects	Preclinical Model	Reference
Decreasing	VAS3947	NOX2i ^1^	HMCL	[81]
Increasing	APR-246	GSH depletion	HCML Primary cell In vivo	[163]
	CAPE	GSH	HCML	[166]
	APR-246/BSO	GSH depletion/γGCSi	In vivo	[161]
	APR-246/auranofin	GSHdepletion/TXNRD1i r	HCMLs Primary cells	[164]
	Auranofin	TXNRD1i	HCMLs	[167]
	Auranofin/ZnPP IX	TXNRD1i/HO1i	HCMLs	[127]
	PX-12	TXNi	HCMLs	[168]
	Auranofin/BTZ	TXNi/PI	HCMLs	[81]
	Lenalidomide/BTZ	TXNi/PI	HCMLs	[169]
	LCS-1	SOD1i	HCMLs Primary cells In vivo	[130]
	2-methoxyestradiol/BTZ	SOD2i/PI	HCMLs	[129]
	Disulfiram/BTZ	SOD1i/PI	HCMLs	[128]
	Scutellarein/BTZ	SOD2i/PI	HCMLs	[170]
	CCF642/BTZ	PDIi/PI	HCMLs In vivo	[171]
	E64FC26	PDIi	HCMLs In vivo	[172,173]
	L-asparaginase/CFZ	AA depletion/PI	HCMLs	[174]
	DPI/HK2-ASO/PER	Mitochondria complex I/HK2i/FAOi	HCMLs In vivo	[175]
	SR18292	PCG-1αi	HCMLs Primary cells	[176]

^1^ Abbreviations: AA, amino acid; ASO, antisense oligonucleotide; BTZ, bortezomib; CAPE, caffeic acid phenethyl ester; DPI, diphenyleneiodonium; γGCS, γ-glutamylcysteine synthetase; FAO, fatty acid oxidation; HMCL, human cell line; HO1, heme oxygenase 1; i, inhibitor; NOX, NADPH oxidase; PDI, protein disulfide isomerase; PER, perhexiline; PCG-1α, peroxisome proliferator-activated receptor γ (PPARγ) coactivator-1 α; PI, proteasome inhibitor, SOD, superoxide dismutase; TXN, thioredoxin, TXNRD1, thioredoxin reductase 1; ZnPP, zinc protoporphyrin.

### 4.2. Drugs That Target Antioxidant Enzymes

MM cells display increased expression of TXN and TXNRD1 involved in the protection against oxidative stress. Moreover, antioxidant genes are overexpressed in myeloma patients, including those with a poor prognosis, and MM cell lines when compared to normal plasma cells [81]. As expected, the targeting of antioxidant enzymes could be beneficial for MM patients.

#### 4.2.1. Thioredoxin System Inhibitors (Auranofin and Other Gold Compounds)

As described before, the thioredoxin system comprising TXN and TXNRD1 is one of the major antioxidant systems in MM cells. In turn, TXN and TXNRD1 inhibition results in ROS-induced apoptosis [125,168]. Gold compounds have a high affinity for thiol and selenol groups and auranofin is very efficient to induce MM cells death, including PI-resistant cells and those with p53 deficiency [81,125,164,167,168]. TXN inhibition activates mitophagy, the selective degradation of mitochondria by autophagy, and negatively regulates the AKT/mTOR and ERK1/2 survival signaling pathways [168]. TNXRD1 inhibition impacts the NFκB signaling pathway [167]. Importantly, auranofin combined with BTZ alleviates the chemoresistance mediated by the tumor microenvironment [81]. A bis-chelated tetrahedral gold(I) phosphine complex seems even more powerful than auranofin to induce ROS-mediated apoptosis [177]. However, TNXRD1 inhibition could be compensated by the overexpression of HO1 through the NRF2 signaling pathway [127]. HO1 pharmacological inhibition using zinc protoporphyrin IX restores BTZ sensitivity [127]. A number of drugs targeting TXN and TXNRD1 and inducing ROS, have been described in the past decade [12], but their ability to synergize with PI in MM patients remains to be established. Nevertheless, the targeting of multiple antioxidant systems could be essential for an efficient anti-MM strategy.

The IMiDs, lenalidomide and pomalidomide, are thalidomide analogs. They inhibit TXNRD1 that leads to an accumulation of cytotoxic H_2_O_2_ levels [169]. In contrast with auranofin and gold compounds, the cytotoxicity of IMiDs necessitates cereblon (CRBN), the substrate receptor the CUL4-RING E3 ligase complex [178]. Indeed, IMiDs/CRBN complexes are retained in the cells and change the redox status by inhibiting H_2_O_2_ degradation. Thus, MM cells with a low antioxidant capacity display increased sensitivity to IMiD-mediated cell death [178].

#### 4.2.2. Superoxide Dismutase (SOD1/2) Inhibitors

Both SOD1 and SOD2 enzymes are overexpressed in MM cells and cell lines compared to normal plasma cells and mediate BTZ-resistance [81,128,129,130]. The inhibition of SOD1 with disulfiram and SOD2 with 2-methoxyestradiol induces apoptosis of both BTZ-sensitive and -resistant MM cells [128,129]. Among the various mechanisms of BTZ resistance, the 19S associated-ubiquitin receptor Rpn13 plays a major role since its inhibition restores BTZ sensitivity [179]. SOD1 is the mediator of Rpn13 signaling and in turn, SOD1 inhibition using the LCS-1 inhibitor, induces a ROS-mediated MM cell death including BTZ-resistant cells [130]. Scutellarein, a flavone extracted from a traditional Chinese medicinal herb, induces a mitochondrial-mediated apoptosis in the MM cells by activating SOD2. This leads to ROS accumulation and synergistic effects combined with BTZ [170].

### 4.3. Other ROS-Inducing Drugs

PDIs are ER-resident oxidoreductase enzymes that ensure the proper folding of nascent polypeptide chains by forming bonds between cysteine residues. PDIs are overexpressed in MM [172]. The inhibition of PDIs induces the accumulation of misfolded or unfolded proteins, an ER stress, and an oxidative stress. Moreover, both pathways are enhanced in response to PIs [171,172,173]. In turn, the concomitant inhibition of PDIs and UPR further enhances the proteotoxic/ER stress and oxidative stress, and the apoptotic response.

Amino acid depletion triggered by L-asparaginase (ASNase) after hydrolysis of glutamine (glutaminolysis) sensitizes MM cells to CFZ [174]. This occurs via NRF2 upregulation, increased mitochondrial ROS generation and mitochondrial dysfunction. Deregulating the protein and amino acid synthesis programs allows PI-resistant MM cells to enter apoptosis [174].

OXPHOS genes are often overexpressed in MM cells and associated with a poor prognosis [176]. This is due to the overexpression of the transcriptional coactivator peroxisome proliferator-activated receptor gamma coactivator-1α (PGC-1α). In turn, OXPHOS genes are enriched in MM patients with high PGC-1α level and the PGC-1α inhibitor ST18292 exerts efficient antimyeloma effects trough ROS production [83].

## 5. Global Alteration of MM Metabolism

More generally, several metabolic pathways are altered in MM cells offering a plethora of potent targets (Figure 3) [83]. MM cells are particularly dependent on glycolysis. GLUT1/4 glucose transporters are expressed on MM cells and their activity is necessary for cell survival [180,181]. Ritonavir, a HIV protease inhibitor reversibly binds GLUT4 and induces MM cell apoptosis [132]. HK2, the first rate-limiting enzyme of the glycolysis cascade is expressed on MM cells at diagnosis and its overexpression is associated with poor prognosis [110,175]. HK2 converts glucose into G6P. The targeting of HK2 by the small molecule 3-bromopyruvate (3-BP) is efficient for killing MM cells [182]. Interestingly, the knockdown of HK2 by an antisense oligonucleotide (ASO) associated with DPI or metformin as mitochondrial complex I inhibitor, and perhexiline as β-oxidation inhibitor induces specifically tumor cell death [175]. The targeting of ENO1 that mediates the conversion 2-phospho-D-glycerate to phosphoenolpyruvate at the final step of the glycolytic pathway, also induces MM cell death. ENO1 is overexpressed in MM samples [183,184]. Other glycolytic enzymes such as phosphofructokinase (PFK), pyruvate kinase M2 (PKM2), and lactate dehydrogenase (LDHA) are also highly expressed in MM cells becoming attracting targets [110,137].

Significant amounts of lactate are produced in MM cells confirming that the oxidative glycolysis is fully functional, but lactate is also produced by the stromal cells within the TME. Lactate enters MM cells through the MCT1 transporter and fuels OXPHOS [134]. The inhibition of MCT1 by the α-cyano-4-hydroxycinnamic acid reduces lactate incorporation and causes apoptosis [134]. Recently, phosphatase of regenerating liver 3 (PRL3), an interleukin-6-induced oncogenic phosphatase, has been shown essential for the metabolic changes of myeloma cells. PRL3 exerts pleiotropic effects. It promotes glucose uptake and glycolytic flux through the upregulation of glycolytic enzymes, and regulates the SSP leading to an increase in GSH intracellular content [184]. PRL3 is highly expressed in MM cells and correlates with poor prognosis and unfavorable outcome [185].

As much as glucose, glutamine is used as an energy provider and MM cells are addicted to glutamine [139]. Alanine/serine/cysteine-preferring transporter 2 (ASCT2), L-type amino acid transporter (LAT1), and sodium-coupled neutral amino acid transporter 1 (SNAT1) are the glutamine transporters expressed on MM cells [139]. Although ASCT2, LAT1, and SNAT1 inhibitors have been assessed on several types of tumors, to our knowledge there is no data on myeloma. Glutamine synthase (GS) is lacking in MM cells, therefore MM cells are dependent on glutamine uptake. By contrast, glutaminase 1 (GLS1) expression is increased [139]. In turn, the inhibition GLS1 that catalyzes glutamine, by benzophenanthridinone (BPI) induces MM apoptosis [186]. An increased glutamine anaplerosis toward TCA cycle is observed in malignant MM cells and this increase is even more marked in comparison with MGUS and overt myeloma [187]. Importantly and different from normal plasma cells in this regard, glycolysis and OXPHOS compensate each other as well as glycolysis and glutaminolysis [132].

Metabolic reprogramming in MM cells is also necessary for cells to adapt their TME. We have previously seen that glutamine, lactate, and probably other metabolites enter MM cells from the TME and in turn, change profoundly the cell metabolism. Furthermore, mitochondria are trafficking between MM cells and mesenchymal stromal cells through tumor-derived nanotubes [141]. The enhanced OXPHOS of MM cells could be the outcome of massive mitochondria acquisition and metabolism reprogramming. Although NOX2 could drive mitochondria transfer from stromal cells to tumor cells as shown for leukemic blasts [188], we have reported that a pan-NOX inhibitor induces MM cell death but shows adverse effect when combined with BTZ [81]. Alternatively, NOX inhibitors could be associated with drugs that have no impact on ROS production rather modifying glycolysis or glutaminolysis. Hypoxia is also a hallmark of the bone marrow niche and HIF-1α and HIF-2α that are stabilized in MM cells control glycolysis through the upregulation of genes coding for glycolytic enzymes and redox homeostasis.

The vulnerability of MM cells to fatty acid metabolism is less studied. Fatty acid synthase (FASN) is overexpressed in MM patients and the inhibition of β-oxidation as well as de novo fatty acid synthesis induces MM cell death including BTZ-resistant cells [189]. Disruption of fatty acid oxidation confers sensitivity to CFZ [94]. Moreover, PI-resistant MM cells increase lipogenesis as a mechanism of resistance [19]. Perhexiline (PER) as a β-oxidation inhibitor synergizes with HK2-antisens oligonucleotide (ASO) for inducing cell death. Cell death is even dramatically enhanced with the triple combination PER/DPI/HK2-ASO [175]. Obesity is a risk factor for MM and bone marrow-resident adipocytes sustain MM growth [190]. By analogy with leukemias, a metabolic shift from glycolysis to enhanced β-oxidation could impact on MM survival [191].

Interestingly, diet and nutrition are linked to risk factors for multiple myeloma [192,193]. Dietary factors may affect inflammation and endogenous growth factors pathways (e.g., IL6, insulin-like growth factor) thereby playing an important role in MM pathogenesis and in patients’ survival [194]. Moreover, diet may also impact the risk of developing MGUS, the premalignant condition of MM [195].

The growth of MM cells within their bone marrow niche necessitates a metabolic shift that shapes the TME toward a hypoxic, acidic, and nutrients-poor milieu [196]. In turn, TME becomes unfavorable for immune cells including T cells and NK cells to exert their antitumor effects. TME displays also tumor-promoting activity by allowing the polarization of M2 macrophages and inhibiting regulatory T cells (Tregs). Several recent reviews are dedicated to this theme [148,196,197,198], we rapidly report here some important clues. The PD-1/PD-L1 pathway controls, at least in part, the maintenance of immune surveillance within the TME [199]. PD-L1 expression is increased in MM cells and associated with disease progression. Although several regulatory pathways are involved in the regulation of PD-L1 level such as JAK/STAT, PI3K/AKT, and ERK/MAPK pathways [200], it is worth noting that HIF-1α directly regulates positively *CD274* gene (encoding PD-L1) transcription [201]. The increased expression of PD-L1 increases cell resistance to cytotoxic T-cell-mediated lysis [202]. Other immune-suppressive cells such as MDSCs [203] and TAMs [204] express high levels of PD-L1. One can imagine that the presence of hypoxic niches in the bone marrow drives the stabilization of HIF-1α that favors PD-L1 expression also in these cells. Moreover, hypoxia inhibits the killing potential of NK cells favoring immune evasion [205]. As described for melanoma cells, the acidity within the TME imposed by an enhanced glycolysis could impact tumor infiltrating lymphocytes (TILs) in promoting anergy and macrophages polarization supporting tumor growth [206,207]. These immunosuppressive pathways should be considered for efficient anti-myeloma therapy.

## 6. Autophagy and Ferroptosis Are Forms of Death Controlled by MM Metabolism

Due to their function of immunoglobulin synthesis, MM cells are addicted to UPS and to another protein degradation pathway, macroautophagy referred to as autophagy [208]. Autophagy is the cell mechanism of self-destruction for clearing organelles and compensate proteasome deficiency to degrade ubiquitinylated proteins. SQSTM1 is the autophagy cargo receptor that bridges ubiquitinylated proteins and autophagosomes and is a critical mediator of autophagy and PI-resistance [209]. In BTZ- and CFZ-resistant cells, SQSTM1 is overexpressed through the activation of NRF2 and this affects the fatty acid oxidation and in turn the level of NADPH [94]. The simultaneous inhibition of UPS and autophagy by hydroxychloroquine induces a synergistic cytotoxicity [210]. Furthermore, *FAM46C* that encodes a non-canonical poly(A) polymerase is mutated in almost 20% of MM patients. FAM46C sustains ER biogenesis in MM cells and this activity is controlled by autophagy through an inhibitory interaction with SQSTM1 [211]. This SQSTM1/FAM46C interplay could be exploited to increase PI efficacy and alleviate PI resistance.

Another type of cell death has been described recently for MM cells: ferroptosis [212]. Ferroptosis is an iron-dependent programmed cell death characterized by a high lipid peroxidation and accumulation of ROS that occurs mainly through intracellular GSH depletion and decreased activity of GPX4 [213]. One way to reduce GSH level is to inhibit the Xc- antiporter that allows cysteine to enter cells (Figure 3). Starheim and coworkers have reported previously that inhibiting Xc- activity induces MM cell death including BTZ-resistant cells. However, in this report the nature of cell death was not examined. GPX4 is a key regulator of ferroptosis inhibiting the formation of lipid peroxide. GPX4, highly expressed in MM cells, is targeted by FTY720 leading to MM ferroptosis [212]. It appears that autophagy and apoptosis play a role in the occurrence of ferroptosis [213]. Although such interplay has not been reported for MM cells, it opens new perspectives for combinatory therapies.

## 7. Conclusions

MM cells have increased ROS levels in comparison to plasma cells, their normal counterparts. This high level of ROS contributes to the initiation, promotion, and progression of MM disease, as well as, resistance to chemotherapy. Therefore, increasing ROS to highly toxic levels may provide a unique tool to kill myeloma cells. This approach seems very efficient since ROS-inducing agents co-operate with PIs (and probably other drugs) and induce MM cell apoptosis including those that are PI-resistant. However, to our knowledge, despite preclinical evidences, no clinical trials using either drugs targeting antioxidant enzymes or depleting glutathione are currently ongoing (www.clinicaltrials.gov). One main concern is to know what are the effects of pro-oxidants on the MM cancer-stem cell (CSC). MM-CSCs is a rare subpopulation of cells that has the capacity for self-renewal and tumor initiation. CSCs are responsible for drug resistance and disease relapse [214]. Although the phenotype of MM CSC is still debated, by analogy with other hemopathies and solid tumors, CSCs likely, synthesize low levels of ROS and in turn, are little impacted by pro-oxidants. Indeed, pro-oxidants inhibit tumor cells proliferation but may have limited impact on cancer-stem cells that synthesize low ROS levels [215]. Moreover, in the perspective of an anti-myeloma immunotherapy, the complex network of immune cells, non-immune cells, and MM cells in their niche, including their redox status, should be better understood.

Interestingly, several unrelated drugs are acting by modulating the redox status. Melphalan is an alkylating agent; its toxicity is due to a redox imbalance and melphalan resistance is linked to modulation of metabolic pathways as well as oxidative stress [216,217,218]. The depletion of intracellular GSH by BSO significantly enhances melphalan activity [216,217]. The next-generation melphalan pro-drug melflufen (melphalan flufenamide ethyl ester) is an alkylating agent that has shown its high efficacy. It was recently approved by the FDA for use in combination with dexamethasone in R/R MM patients [219]. Melflufen enters cells where it is targeted by aminopeptidases resulting in its trapping in organelles and membranes allowing its accumulation. In pre-clinical models, it has been shown that it generates ROS [220]. HDAC inhibitors, which regulate gene expression by inhibiting the deacetylation of histone proteins, have been shown to exert a wide array of antitumor effects including cell cycle arrest and cell death by generating ROS [221,222].

Metabolism changes in MM cells show their vulnerability. For efficient killing of myeloma tumor cells, the design of metabolic targets and the choice of new combinations must take into account the modifications of metabolism imposed by the TME as well as the redox status of MM cells themselves.

## Abbreviations

3PG, 3-phosphoglycerate; 3-PS, 3-phosphoserine; 6-PG, 6-phosphogluconate; αKG, alpha-ketoglutarate; AP1, activator protein 1; ARE, antioxidant response element; ASCT, amino acid exchanger; ATF, activating transcription factor; ATP, adenosine triphosphate; BSO, L-buthionine sulfoximine; BTZ, bortezomib; b-ZIP, basic leucine zipper; CAT, catalase; CBP, CREB binding protein; CFZ, carfilzomib; COX, cyclo-oxygenase; CRBN, cereblon; CREB, cAMP-response element binding protein; CUL, cullin; DPI, diphenyleneiodonium; EGF, epidermal growth factor; ENO1, α-enolase; ER, endoplasmic reticulum; ERK, extracellular signal-regulated kinase; ERO1, endoplasmic reticulum oxidoreductin; ETC, electron transport chain; ETFDH, electron transfer flavoprotein dehydrogenase; FASN, fatty acid synthase; G6P, glucose-6-phosphate; G6PD, G6P, glucose-6-phosphate dehydrogenase; GCL, glutamate-cysteine ligase or γ-glutamylcysteine synthetase; GLRX, glutaredoxin; GLS, glutaminase; GLUT, glucose transporter; GPD2, glycerol-3-phosphate dehydrogenase 2; GPX, glutathione peroxidase; GSH, reduced glutathione; GSR, glutathione reductase; GSSG, oxidized gltathione; HDAC, histone deacetylase; HIF, hypoxia-inducible factor; HO1, heme oxygenase 1; HSF1, heat-shock factor 1; IMiD, immunomodulator; JAK, Jason kinase; JNK, c-Jun N-terminal kinase; KLF, Krüppel-like factor; LAT, L-type amino acid transporter; LDHA, lactacte dehydrogenase A; LOX, lipoxygenase; MAF, musculoaponeurotic fibrosarcoma; MAPK, mitogen-activated protein kinase; MCT, monocarboxylate transporter; MEK, mitogen-activated protein kinase kinase; MM, multiple myeloma; mTOR, mammalian target of rapamycin; MUC1, mucin 1; NAD, nicotinamide adenine dinucleotide; NADPH, nicotinamide adenine dinucleotide phosphate; NFκB, nuclear factor NADH kappa-B; NOS, nitric oxide synthase; NOX, NADPH oxidase; NRF2, nuclear factor-erythroid 2 p45-related factor 2; OGDH, 2-oxoglutarate dehydrogenase; OXPHOS, oxidative phosphorylation; PDC, pyruvate dehydrogenase complex; PDGF, platelet-derived growth factor; PDK, pyruvate dehydrogenase kinase; PFK, phosphofructokinase; PGC-1α, PPARγ-coactivator 1-α; PHGDH, phosphoglycerate dehydrogenase; PHD, pyruvate dehydrogenase; PHGDH, phospho-glycerate dehydrogenase; PI, proteasome inhibitor; PKM1/2, pyruvate kinase isoform M1/2; PPARγ, peroxisome proliferator-activated receptor γ; PPP, pentose phosphate pathway; PRDX, peroxiredoxin; PRL3, protein-tyrosine phosphatase of regenerating liver 3 or PTP4A3; PRIMA-1, p53 reactivation and induction of massive apoptosis-1; R, receptor; PSAT1, phosphoserine aminotransferase 1; PSPH, phosphoserine phosphatase; R5P, ribulose-5-phosphate; R/R, relapse/refractory; ROS, reactive oxygen species; SHTM1/2, serine hydroxymethyltransferase 1/2; SNAT, sodium-coupled neutral amino acid transporter; snoRNA, small nucleolar RNA; SOCS, suppressor of cytokine signaling, SOD, superoxide dismutase; SQSTM1, sequestosome 1 or p62; SSP, serine synthesis pathway; STAT, signal transducer and activator of transcription; TACI, transmembrane activator and calcium-modulating cyclophilin ligand interactor; TCA, tricarboxylic acid; TIGAR, TP53-inducible glycolysis and apoptosis regulator; TK, tyrosine kinase; TME, tumor microenvironment; TNF, tumor necrosis factor; TRAF, TNF receptor-associated factor; TXN, thioredoxin; TXNRD1, thioredoxin reductase 1; UPR, unfolded protein response; UPS, ubiquitin/proteasome pathway; VHL, Von Hippel-Lindau tumor suppressor; XDH, xanthine oxidase.
